# Factors Predicting the Intention of Eating an Insect-Based Product

**DOI:** 10.3390/foods8070270

**Published:** 2019-07-19

**Authors:** Simone Mancini, Giovanni Sogari, Davide Menozzi, Roberta Nuvoloni, Beatrice Torracca, Roberta Moruzzo, Gisella Paci

**Affiliations:** 1 Department of Veterinary Sciences, University of Pisa, Viale delle Piagge 2, 56124 Pisa, Italy; 2 Department of Food and Drug, University of Parma, Parco Area delle Scienze 27/A, 43124 Parma, Italy; 3 Interdepartmental Research Center Nutrafood “Nutraceuticals and Food for Health”, University of Pisa, Via del Borghetto 80, 56124 Pisa, Italy

**Keywords:** entomophagy, novel food, neophobia, disgust, edible insects

## Abstract

This study provides a framework of the factors predicting the intention of eating an insect-based product. As part of the study, a seminar was carried out to explore how the provision of information about ecological, health, and gastronomic aspects of entomophagy would modify consumer beliefs regarding insects as food. Before and after the informative seminar, two questionnaires about sociodemographic attributes and beliefs about the consumption of insects as food were given. Participants were then asked to carry out a sensory evaluation of two identical bread samples, but one was claimed to be supplemented with insect powder. Results showed that perceived behavioral control is the main predictor of the intention, followed by neophobia and personal insect food rejection. The disgust factor significantly decreased after the participants attended the informative seminar. Sensory scores highlighted that participants gave “insect-labelled” samples higher scores for flavor, texture, and overall liking, nevertheless, participants indicated that they were less likely to use the “insect-labelled” bread in the future. Our findings provide a better understanding of insect food rejection behavior and help to predict the willingness to try insect-based products based on some important individual traits and information.

## 1. Introduction

In Europe, the habit of eating insects, or entomophagy, has not become widespread, with the exception of some specific and local cases [1,2]. Traditionally, most people in Western countries consider insects to be a threat and a health risk rather than a food source [3], despite the fact that insect protein and fat contents, mineral contents, and amino acids profiles confirm that they are a good source of nutrients [4].

Most of the studies carried out in Europe about consumers’ acceptance of insects as food illustrate a low consumer willingness to try eating insects [5], with the exception of the Netherlands, Belgium, and few other Northern countries, where some insect-based products have been commercially available in the past few years [6,7,8]. Particularly, in urban and Western societies, insects are rarely eaten or their consumption is perceived to be culturally inappropriate [2]. As reported by Deroy et al. [9], one of the greatest barriers to adopting entomophagy is cognitive disgust linked to the perception that insects are related to undesirable thoughts such as dirt, death, disease, and contamination [10]. Only a few works have investigated how potential consumers from Southern European countries might respond to entomophagy [11].

The theory of planned behavior (TPB) [12], has been proven to be a proper theoretical framework for understanding sustainable and ethical consumer behaviors concerning food [13,14]. According to TPB, the perceived behavioral control (PBC), along with subjective norms (SN) and attitude, impact a person’s intention. Perceived behavioral control is the measure of perceived control over the behavior (i.e., how easy or difficult performing the behavior will be). Perceived behavioral control can also have a direct impact on behavior because performance of a behavior not only depends on motivation, but also the individual control of the behavior. If an individual has limited control over an activity, the activity might not be implemented, even in the presence of strong motivational factors.

Menozzi et al. [15], examined whether the TPB could be employed to understand young adults’ behavior when faced with the prospect of eating products containing insect flour, and suggested that attitude and, to a lesser extent, PBC play a significant role in affecting the intention of performing the behavior, while the SN is not a significant factor in forming the behavioral intention. This result is common in several other studies [16], and the SN is generally a weak predictor of intentions [17].

From another point of view, when a new food product is introduced into a culture, it generally induces feelings of fear and refusal that is called food neophobia [18], which refers to the unwillingness to try and the tendency to avoid novel food [19].

Food neophobia is expected to reduce the likelihood of readiness to incorporate insects into the diet [20,21,22]. Pliner and Hobden [19] developed an instrument to measure food neophobia which consisted of ten statements, five positively worded (neophilic) and five negatively worded (neophobic), and was called a food neophobia scale (FNS).

Recently, many studies have indicated that food neophobia, as an individual trait, is one of the most important predictors for understanding consumer willingness to try insects [22,23,24,25]. The relationship between entomophagy and food neophobia may depend on the knowledge of their origin and habitat or may be based on negative consequences following their consumption [26].

Food exposure increases product familiarity and influences the willingness to accept new food, and thus reduces neophobic reactions [21,27,28,29]. At the same time, information is a factor that plays a role in consumers’ acceptance [30,31] and can be used to promote entomophagy [22]. Information about insects, such as production technology, safety risk, and scientific-like information [32,33], tailored to consumers can modify their perception of items, both novel and familiar.

Despite the huge interest in factors specifically related to the intention of eating insects, there is a lack of research that investigates the link between the mechanism of rejection of insects as food (both at a personal and social level), the level of food neophobia for unfamiliar food, and the perceived behavior control of introducing insects to the diet.

On the basis of these assumptions, the first aim of this study was to evaluate the willingness of consumers to adopt insects as food and to investigate the main factors (e.g., sociodemographic variables, food neophobia, and other behavioral constructs) that affect the intention to eat insects. The second aim was to investigate whether and how information can influence the willingness to taste insects and their acceptability. This was achieved by comparing consumers’ willingness to adopt insects before and after a seminar on insects, and investigating how an informative seminar on the benefits of including insects in human diets influenced the consumer acceptance. In addition, an analysis about the perception of the sensory properties of two identical breads (“insect-labelled” vs. “no insect-labelled”) was performed.

## 2. Material and Methods

### 2.1. Theoretical Model

The model includes two concepts, food neophobia and insect food rejection, with the introduction of some elements of the TPB.

**Figure 1:**
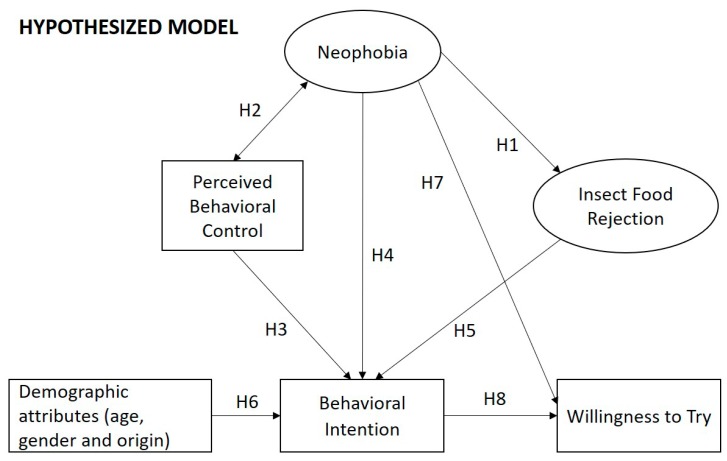
The conceptual model corresponding to the current research. Our study tested the following hypotheses (H): H1, food neophobia will positively affect rejection factors of insect as food (e.g., disgust) [10]; H2, food neophobia and the perceived behavioral control (PBC) will be correlated; H3, PBC will have a significant positive effect on the intention of eating an insect-based food in the coming months [15]; H4, a higher level of food neophobia will have a strong negative effect on the behavioral intention of eating an insect-based food in the coming months [21]; H5, a higher level of insect food rejection will have a strong negative effect on the intention of eating an insect-based food in the coming months; H6, demographic attributes will have a moderate effect on the intention of eating an insect-based food in the coming months [34]; H7, food neophobia will negatively affect the willingness to eat an insect-based product; H8, intention of eating an insect-based food in the next months will be a strong predictor of the willingness to eat an insect-based product [15].

In accordance with previous studies, given the premises mentioned in the introduction, we tested the following hypotheses (Figure 1).

### 2.2. Participants and Data Collection

The survey was conducted at the University of Pisa (Italy) in March 2018. Participants were students attending a seminar entitled “Insects as Food and Feed: Future Prospects”. The three-hour seminar was structured as oral presentations from different speakers with visual support. The aim of the seminar was to describe benefits, disadvantages, and future prospects of insects as food and feed. In particular, aspects were delivered related to species and breeding technologies, food safety and regulatory concerns, and consumers’ approach.

A total of 165 students with a bachelor’s degree and a master’s degree participated in this study. They did not receive monetary compensation for their participation. Prior to the seminar session, participants were informed about the questionnaires and an informed consent form was signed.

Our protocol was based on previous studies where an informative session about entomophagy was held among university students followed by an insect tasting session [34,35]. Two questionnaires were proposed chronologically to the students: one before (questionnaire 1, Q1) and another after (questionnaire 2, Q2) the seminar. Furthermore, at the end of Q2, the participants were invited to take part in a tasting session immediately after the seminar ended.

### 2.3. Questionnaire Design and Measures

The structure of the questionnaire for both Q1 and Q2 was based on previous study designs [15,34]. The first part of the questionnaire (Q1) included three questions concerning the demographic profile of the respondents (i.e., age, gender, and Italian region of origin) and 20 individual statements, divided into two different sections. Section A included 10 items about a person’s tendency to reject or avoid eating unfamiliar food (neophobia). Section B, which followed A, included 10 items focused on beliefs towards the consumption of insects (i.e., disgust, distaste, fear, and social acceptance). For all the statements, responses were given on a seven-point agreement scale, ranging from ‘‘1 = strongly disagree’’ to ‘‘7 = strongly agree’’.

In addition, participants were asked whether they had ever eaten insects, and if yes, they were asked to indicate on what occasion and their liking of the experience (i.e., “If you have already tried eating insects in the past, how much did you like it?”). Next, they were asked to evaluate the importance of possible barriers to the consumption of insects (e.g., negative taste and texture, low level of food safety, being vegetarian).

The second questionnaire (Q2) was composed of three sections. The first section was composed of three items that measured behavioral beliefs toward eating products containing insect powder. The aim was to evaluate whether this practice would have: (1) “positive effects on health”, (2) “positive effects on the environment”, and (3) “a familiar taste compared to known products”. The second part of the questionnaire was structured in the same way as section B of Q1 in order to compare the participants’ beliefs before and after the informative seminar. The last part included the intention of eating insect-based food in the coming months (behavioral intention) and the perceived behavioral control, i.e., “I believe that eating products containing insect powder in the coming months is possible.” Finally, participants were asked about their willingness to eat insects (i.e., whether they wanted take part in the tasting session). If participants decided to not attend the tasting section, they were asked why (e.g., short of time, afraid to being allergic, afraid of health risks, vegetarian or other).

### 2.4. Tasting Session

Although the students who agreed to take part in the tasting session had been told that edible insects would be included, no further detail was given about what food they were going to taste at the end of the session, as suggested by Spence [36]. Participants were asked to score five sensory attributes of each sample (appearance, odor, flavor, texture, overall liking) using a nine-point balanced hedonic scale (1, extremely negative; 5, neither negative nor positive; 9, extremely positive) and to express the probability of consuming the product in the future using a similar nine-point scale (1, extremely improbable; 9, extremely probable). The two bread samples were identical, except one was claimed to be supplemented with insect powder, i.e., “insect-labelled” bread, although it did not contain any insect ingredients. Bread was chosen as the food product because consumers were familiar with it, an attribute which has been suggested to reduce food neophobia among Western consumers [23,27,35]. The tasting session was organized in a separate room, and once they had tasted the bread samples, participants were asked to not share information or impressions with others that had not yet tried them. Participants were also informed about the safety of the bread preparations and the allergenic potential of insects, even though the “insect-labelled” bread did not contain insect.

The two types of samples came from the same pre-sliced wholemeal bread (Lidl Stiftung & Co. KG, Neckarsulm, Germany), and they were the same size (5.7 cm × 5.7 cm × 1.1 cm, 9 g), measuring one quarter of a whole slice.

Samples were presented monadically in white plastic dishes, and the samples of “insect-labelled” bread were clearly indicated to the participants. Participants were instructed to drink water between sample tasting to neutralize the taste. Ordering of samples was randomized, so that approximately half of participants tasted the “control” bread first while the other half tasted the “insect-labelled” bread first (32 and 34 participants, respectively).

### 2.5. Statistical Analyses

A comparison of mean scores, i.e., independent samples *t*-test, was used to assess associations between behavioral beliefs about eating products containing insect powder (interval variables, seven-point scale) and willingness to try an insect-based food (dummy variable: yes or no).

Additionally, correlational analyses (Pearson correlation) between behavioral beliefs and behavioral intention to eat a product containing insect powder in the coming months was conducted.

Exploratory factor analyses (EFA), principal component extraction method, and varimax (orthogonal) rotation, were run to assess the unidimensional structure of the food neophobia and food rejection constructs [37]. The Cronbach’s alpha was used to assess the internal consistency of the construct scales, where a value greater than 0.7 is usually recommended. Finally, a structural equation modelling (SEM) technique was employed on the data to test for the model identified in Figure 1. The SEM allows for the specification of model structure with both latent and observed variables. Latent variables, i.e., abstract phenomena that cannot be directly measured by the researcher, were analyzed using confirmatory factor analysis (CFA). CFA, often referred to as the measurement model, is used to test for the underlying latent variable structure. The statistical significance of parameter estimates was tested with the critical ratio (C.R.), i.e., the parameter estimate divided by its standard error. It operates as a z-statistic for testing that the estimate is statistically different from zero [37]. The model fit of both CFA and SEM was assessed using the chi-square (χ^2^), the comparative fix index (CFI), the Tucker–Lewis index (TLI), the standardized root mean square residual (SRMR), and the root mean square error of approximation (RMSEA) with 90% confidence. Since the chi-square is affected by sample size, the application of multiple fit indices is usually recommended. An acceptable fit based on these indexes is considered with CFI and TFI ≥0.9 and RMSEA ≤0.08. The width of the RMSEA confidence interval is informative about the precision in the estimate of the RMSEA. For SRMR a value less than 0.08 is generally considered a good fit [38,39]. The coefficient of determination (R^2^) measured the explained variance of the endogenous variables (intention and willingness to try). We applied the Bayesian estimation routine in IBM^®^ SPSS^®^ AMOS 24.0 (IBM, Armonk, NY, USA) recommended for analyzing categorical data [37].

The statistical difference of the correlation between attendance at the tasting session and gender of participants was tested using the chi-squared test of independence. Generalized linear model (GLM) statistics was used on the tasting session scores to evaluate the significance of the bread sample type, gender, and tasting order effects. Moreover, for each participant, delta scores were calculated subtracting for each evaluated parameter the score of the “control” bread from the score of the “insect-labelled” bread; thus, positive deltas indicate a higher score for the “insect-labelled” sample. Then the difference in responses given by each participant to the two types of samples was investigated using a Wilcoxon rank sum test.

## 3. Results

### 3.1. The Influence of Behavioral Beliefs

A two-way ANOVA test was carried out to measure the effects of gender and willingness to try edible insects on the three behavioral beliefs (Table 1) and the relative potential interactions.

No significant potential interactions were found. Surprisingly, no impact of gender was found for any of the beliefs, whereas, the willingness to try had an effect only on the beliefs about the familiar taste of products containing insect powder (4.39 ± 1.11 and 3.97 ± 1.21 for willing and not willing, respectively, *p* = 0.028).

This means that people who did not want to taste the insect-based product have significantly lower scores in the beliefs that the taste is familiar to a conventional product as compared to those who were willing to try them.

In addition, Pearson’s correlation test was performed considering the behavioral intention to eat products containing insect powder in the coming months and the behavioral beliefs. The results of the correlation analysis are all statistically significant. Findings suggest that the belief that eating products containing insect powder will have positive effects on health has a stronger influence on behavioral intentions (0.323, *p* < 0.01) as compared to beliefs about environmental protection (0.235, *p* < 0.01) and familiar taste (0.25, *p* < 0.01).

### 3.2. Post-Seminar

Disgust and the idea of post-consequence of the ingestion, such as negative texture and bad taste, are among the most important barriers to the acceptance of insects as food, whereas, the lowest scores have been assigned to beliefs that entomophagy is not socially acceptable. A *t*-test was conducted to identify the possible differences in strength of the insect food rejection items before and after the seminar session (Table 2). After the informative seminar, the scores of all the items improved, indicating a more positive attitude towards eating insects (i.e., lower rejection). In particular, disgust factor and the fear of negative texture properties were strongly reduced. All these differences, except for the item “I fear that insect-based foods have negative taste properties”, were statistically significant.

### 3.3. Model Testing and Results

The EFA results on the food neophobia construct confirm its unidimensional structure; the Cronbach’s alpha value of 0.86, the overall explained variance (59.4%), the factor loadings, and the communalities all above the 0.5 threshold indicate a good representation and satisfactory internal consistency of the food neophobia construct with this unidimensional structure (Table 3). This structure has also been tested with the CFA, showing a good model fit to the data, i.e., model fit: χ^2^ (5) = 7.002; CFI = 0.996; TLI = 0.987; RMSEA (CI 90%) = 0.049 (0.000, 0.081).

On the other hand, the EFA results suggest that a multidimensional structure of the food rejection construct may be more appropriate. The analysis yielded two factors explaining a total of 70.4% of the variance for the entire set of variables (Table 4).

The first factor, explaining 52.8% of the variance, includes three items related to danger and social inappropriateness of insects as food. This factor was labelled as “social insect food rejection” due to the high loadings by the following items: “I believe that insect-based food implies a poor hygiene”, “I believe that eating insects is not part of our diet”, and “eating insects is not socially acceptable”. The items communalities, representing the proportion of the variance in that variable that can be accounted for by the extracted factors, are all above the 0.5 threshold, indicating that the extracted factors account for a big proportion of the items’ variance. The second factor was labelled “personal insect food rejection” due to the high loadings by the following items: “the idea of eating insects provokes my disgust”, “I fear that insect-based foods have negative texture properties”, and “I fear that insect-based foods have negative taste properties”. This factor explained 17.7% of the variance. Overall, the Cronbach’s alpha of the two factors above the 0.7 value, the factor loadings, and the communalities all above the 0.50 threshold, indicate a good representation and internal consistency of this two-factor model. The CFA was performed to test for this two-factor structure, showing a good model fit to the data (model fit: χ^2^ (8) = 7.207; CFI = 1.000; TLI = 1.004; RMSEA (CI 90%) = 0.000 (0.000, 0.086)).

**Figure 2:**
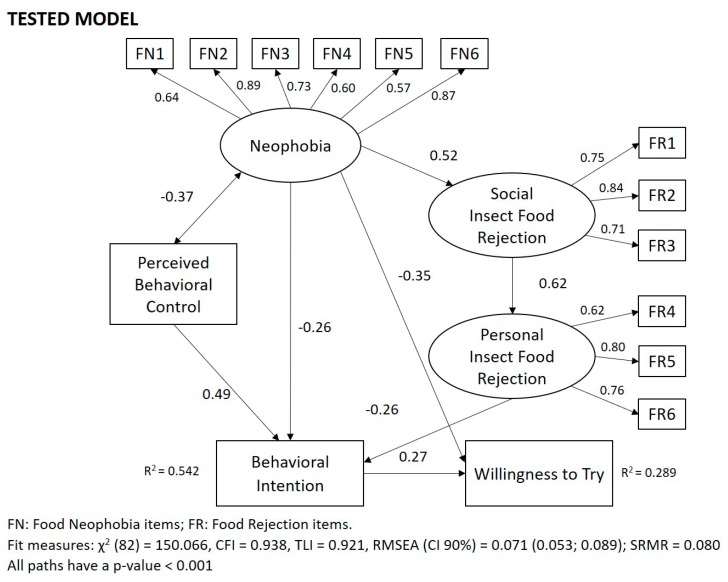
The standardized regression coefficients, explained variance (R^2^), and fit measures.

The prediction of eating insect-based food was examined using structural equation modelling (SEM) techniques. The results are reported in the tested model (Figure 2).

The model fits the data satisfactorily as documented by the fit indices (χ^2^ (82) = 150.066; CFI = 0.938; TLI = 0.921; RMSEA (CI 90%) = 0.071 (0.053, 0.089); SRMR = 0.080) and explains a substantial amount of variance of the behavioral intention (R^2^ = 0.54) and a relative smaller variance of the willingness to eat insect-based food (R^2^ = 0.29).

Confirming H2, a statistically significant negative correlation between neophobia and PBC was detected (*p* < 0.001), suggesting that respondents with lower phobia of new food believe that they can better accomplish the behavior of eating insect-based food and vice versa. PBC is the main predictor of the intention (*p* < 0.001), followed by neophobia (*p* < 0.001) and personal insect food rejection (*p* < 0.001). Therefore, these results confirmed H3, H4, and H5. The willingness to eat insect-based food is negatively affected by food neophobia (*p* < 0.001), and positively influenced by the intention to eat products containing insect powder in the coming months (*p* < 0.001). These results confirmed, respectively, H7 and H8.

Moreover, the results indicate that the social insect food rejection construct mediates the effect of neophobia with personal insect food rejection. In other words, this indicates that a higher neophobia positively affected the perceived social rejection of insects as food which, in turn, influenced the personal rejection. Therefore, the hypothesis suggesting that food neophobia positively influenced factors of the rejection of insects as food (H1) is also confirmed.

Finally, sociodemographic characteristics, such as age, gender, and region of origin, were neither significantly correlated with the intention nor with the willingness to eat insects, and therefore have not been included in the final model. This suggests that H6 was not supported by the data.

Since food disgust sensitivity and neophobia traits can be strongly influenced by familiarity and previous experience, a question about past exposure of insect eating was asked. However, considering the low number of people that had already tasted insects before the study, this question was not included in the model.

### 3.4. Tasting Session

Out of the total 165 participants, 66 (40%) took part in the tasting session; 11 were males and 55 were females, corresponding to 39.29% and 39.86% of total males and females, respectively. As such, no statistical difference was found between the genders in their willingness to participate in the tasting session. The scores of the tasting session are reported in Table 5.

**Figure 3:**
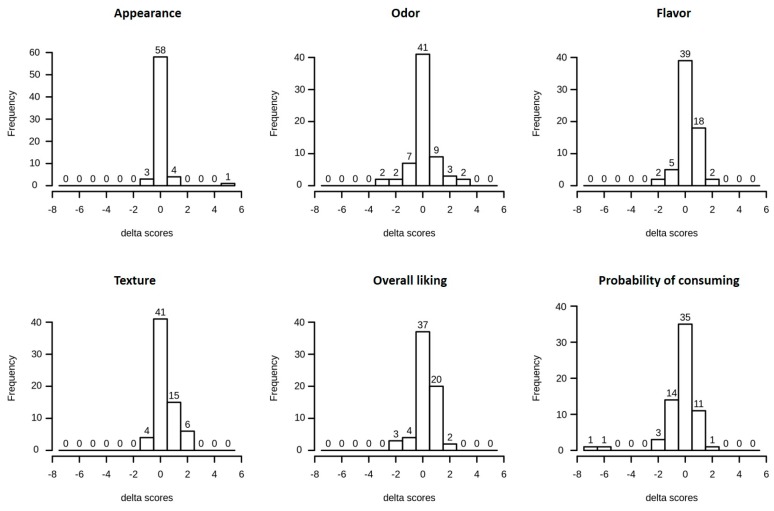
Delta scores between “insect labelled” sample and “control” bread.

The results of the Wilcoxon rank sum test on the sensory scores indicated that participants gave “insect-labelled” samples higher scores for flavor, texture, and overall liking (*p* = 0.044, *p* < 0.001, *p* = 0.045, respectively). This data did not reflect on the score given to the probability of future consuming, since 28.79% of participants indicated that they was less likely to use the “insect-labelled” bread in the future, while only 18.18% stated the opposite (although no statistical difference was found at the Wilcoxon rank sum test). Figure 3 shows the delta scores obtained for each characteristic. Only 13.64% of the participants gave the two bread samples identical scores for every evaluated parameter. In two cases, the delta score for probability of future use was ≤−6 and represented the biggest score difference registered.

## 4. Discussion

### Reactions towards Insects

Our research findings suggest that a variety of factors need be taken into consideration when assessing the willingness to eat insect-based food, from which several implications can be derived both theoretically and practically.

As reported by Hartmann and Siegrist [10], disgust sensitivity prevents consumers from eating harmful substances (e.g., contamination of toxic ingredients), however, it can also become a strong barrier to trying new food and/or unfamiliar food products like insects. The results have shown that the relationship between neophobia and personal factors of insect food rejection is mediated by the social factors of insect food rejection construct, i.e., the perceived danger and social inappropriateness of insects as food. This mediation effect suggests a more detailed relationship between food neophobia and disgust than the one depicted by Hartmann and Siegrist [10]. As expected, neophobic individuals have a higher social rejection (i.e., insects are not part of my diet, not acceptable as food) which is strongly correlated with a greater personal insect rejection (i.e., negative sensory expectations). However, the mediation effect suggests how the personal factors of insect food rejection can be more easily influenced, for instance, by an increasing social appeal of insects as food driven by positive information about entomophagy and the tasting experience. This enhanced the social acceptance of insects as food which may improve the personal factors, and therefore decrease the disgust and distaste. As reported by Barsics et al. [35], long-term exposure to information and positive effects (mainly entomophagy familiarity and experience) could reduce disgust and distance and make better assimilation of the new further information. In addition, other studies reported how information about the sustainability and environmental perspectives of edible insects, could positively influences consumers’ willingness to consume insect-based products [22,30,40,41,42].

According to the literature, since disgust influences people’s food preferences, higher food disgust and distaste sensitivity (personal insect food rejection) are associated with a lower behavioral intention. This has been confirmed in our study, since we have found a significant negative effect of personal insect food rejection on behavioral intentions. Nevertheless, our results also suggest how the insect food rejection construct helps to explain the relationship between neophobia and intention. This is, however, a partial mediation since the direct effect of neophobia on behavioral intention is still significant. The PBC has been found to be the main predictor of the intention, as also suggested by the theory [12].

The willingness to try an insect-based product is positively affected by behavioral intention and negatively influenced by food neophobia. This result is consistent with previous studies [6,10,22,43] indicating that the trait of food neophobia is an important predictor of consumers’ willingness to try insect-based food products. In addition, the positive and significant correlation between behavioral intention and beliefs suggests that consumers believe that this practice will have positive effects on their health and on the environment, as also reported by Sogari [44] and Menozzi et al. [15].

The informative seminar positively influenced all the opinions about entomophagy, improving the consumers’ attitude towards eating insects while lowering insect food rejection. The greatest change after the seminar was found for the disgust factor, which strongly decreased after participants were informed about the technological, social, and cultural context of entomophagy. The communication about the gastronomic aspects of insects influenced only the texture properties and not the taste. This might be explained by the fact that it might be easier to convince people that the texture is similar to known food products (not disgusting) rather than the organoleptic attributes. In addition, the fear regarding poor hygiene decreased following the explanation about the long-time habits of entomophagy around the world and the food safety production practices which will be used before these novel products can be commercialized in the Western countries. Barsics et al. [35] reported that familiarity with entomophagy, intrinsically related with the culture of the consumers, plays a major role in acceptance and a positive attitude toward edible insects. Information could enhance familiarity with entomophagy, nevertheless, it seems more effective if it is continuous with a long-term impact.

The role of communication in decreasing the rejection of new food can be explained by the two hypotheses of Rozin and Fallon [26], who explained this tendency might be related to the origin of the food and/or the anticipated negative post-ingestion consequences. Positive information on eating insects can address these two barriers. Given the strong positive correlations between the positive health effects of eating insects and the behavioral intention, our findings suggest that consumer-targeted communication of health aspects would increase the intention to eat.

Findings of this work have several implications on sensory aspects of product development and the role of information on influencing consumer acceptance.

Our data indicate that the provision of information plays a role in decreasing the rejection of insects as food, both at a personal and social level. To our knowledge, this implication has been previously investigated by only a few other authors [20,35].

We are aware that the likelihood of trying insects will strongly depend on the appropriateness of the preparation method [45,46,47]. If edible insects are to become more widely consumed in the Western countries, positive thoughts and sensory-liking properties need to be associated with familiar products [15,27]. As reported by Schouteten et al. [48], consumers have to associate (positive) emotions with the products (based on the satisfaction of sensory attributes) to replace the negative expected emotions prior to consumption [49].

Although our study used an “insect-labelled” product, the greater sensory appreciation as compared with the similar “no insect” product confirms that integrating insects into the Western diet could be done using a transitional phase [20,50]. Moreover, familiar products could help to increase the likelihood of having the first bite, but to be regularly consumed, they must be appropriate and taste good [47,49].

## 5. Conclusions

Understanding which factors could affect consumers’ perception of edible insects plays a key role in the future prospect of entomophagy and, in general, on novel food protein production and consumption. In summary, the participants’ willingness to eat insect-based products in the coming months appears to be mainly influenced by neophobia and behavioral intention. Communication becomes crucial to achieve this aim and informative tasting sessions are a good approach to influence people to try insect-based foods for the first time. The current study suggests that an educational approach paired with a tasting session could be a good strategy to increase objective knowledge while reducing the insect food rejection mechanism and enhancing the positive evaluation of sensory properties.

## Figures and Tables

**Table 1:** Mean values (standard deviation) of the behavioral beliefs and the effects and interactions with willingness to try (WTT) and gender.

Items	Total Sample		Male		Female		*p*	
	WTT	No WTT	WTT	No WTT	WTT	No WTT	Gender	WTT
Eating products that contain insect powder will have positive effects on my health	4.29 (0.88)	4.18 (0.93)	4.53 (1.06)	4.36 (1.79)	4.24 (0.85)	4.14 (0.91)	0.177	0.426
Eating products containing insect powder will have positive effect on the environment	5.65 (1.13)	5.63 (1.16)	5.63 (1.31)	5.55 (1.03)	5.66 (1.10)	5.65 (1.16)	0.801	0.901
Eating products containing insect powder will have a taste familiar to products that I already know	4.39 (1.11)	3.97 (1.21)	4.38 (0.88)	3.73 (1.00)	4.39 (1.16)	4.02 (1.25)	0.604	0.028

The main effects were reported by removing the interaction terms (gender × WTT) which was not significant for any beliefs.

**Table 2:** Mean values and 95% confidence intervals (CI) for the insect food rejection items, before and after the informative seminar (n = 165).

Items	Before, Mean (sd)	After, Mean (sd)	Mean Difference	95% CI	*p*
The idea of eating insects provokes my disgust	4.80 (1.83)	4.09 (1.79)	0.71	(0.461, 0.957)	0.000
I fear that insect-based foods have negative texture properties	4.61 (1.7)	4.19 (1.61)	0.42	(0.184, 0.653)	0.001
I fear that insect-based foods have negative taste properties	3.99 (1.55)	3.84 (1.59)	0.15	(−0.062, 0.365)	0.164
I believe that insect-based food implies a poor hygiene	3.30 (1.62)	2.99 (1.6)	0.31	(0.065, 0.542)	0.013
I believe that eating insects is not part of our diet	2.91 (1.66)	2.55 (1.44)	0.36	(0.152, 0.575)	0.001
Eating insects is not socially acceptable	2.52 (1.43)	2.27 (1.38)	0.25	(0.042, 0.467)	0.019

Respondents indicated their opinion on a seven-point scale ranging from 1 (“do not agree at all”) to 7 (“totally agree”).

**Table 3:** Confirmatory factor analysis of the food neophobia (FN) construct.

Items		Cronbach’s Alpha	Factor Loadings
Food Neophobia		0.862	
FN1	I am constantly sampling new and different foods (R)		0.613
FN2	I like foods from different cultures (R)		0.909
FN3	Ethnic food looks too weird to eat		0.719
FN4	At dinner parties, I will try new foods (R)		0.568
FN5	I am afraid to eat things I have never had before		0.579
FN6	I like to try new ethnic restaurants (R)		0.874

Model fit: χ^2^ (5) = 7.002; CFI = 0.996; TLI = 0.987; RMSEA (CI 90%) = 0.049 (0.000, 0.081). R-reverse coded.

**Table 4:** Confirmatory factor analysis of the insect food rejection (FR) construct.

Items		Cronbach’s Alpha	Factor Loadings
Social Insect Food Rejection		0.81	
FR1	I believe that insect-based food implies a poor hygiene		0.75
FR2	I believe that eating insects is not part of our diet		0.872
FR3	Eating insects is not socially acceptable		0.684
	Personal Insect Food Rejection	0.758	
FR4	The idea of eating insects provokes my disgust		0.59
FR5	I fear that insect-based foods have negative texture properties		0.816
FR6	I fear that insect-based foods have negative taste properties		0.776

Model fit: χ^2^ (8) = 7.207; CFI = 1.000; TLI = 1.004; RMSEA (CI 90%) = 0.000 (0.000, 0.086).

**Table 5:** Results of tasting section reported as mean (standard deviation).

Items	“control” Sample	“insect labelled” Sample	*p*
Appearance	7.2 (1.32)	7.29 (1.19)	0.407
Odor	6.64 (1.74)	6.7 (1.53)	0.626
Flavor	7.06 (1.12)	7.26 (1.14)	0.044
Texture	6.77 (1.38)	7.12 (1.42)	<0.001
Overall liking	7.18 (1.14)	7.39 (1.09)	0.045
Probability of consuming	7.52 (1.51)	7.21 (1.67)	0.107
